# Correction: Study on photoelectric characteristics of monolayer WS_2_ films

**DOI:** 10.1039/c9ra90088h

**Published:** 2019-11-28

**Authors:** Lin Wang, Wenyan Wang, Quan Wang, Xiaochun Chi, Zhihui Kang, Qiang Zhou, Lingyun Pan, Hanzhuang Zhang, Yinghui Wang

**Affiliations:** Femtosecond Laser Laboratory, Key Laboratory of Physics and Technology for Advanced Batteries (Ministry of Education), College of Physics, Jilin University Changchun 130012 P. R. China yinghui_wang@jlu.edu.cn chixc@jlu.edu.cn; State Key Laboratory of Superhard Materials, College of Physics, Jilin University Changchun 130012 P. R. China

## Abstract

Correction for ‘Study on photoelectric characteristics of monolayer WS_2_ films’ by Lin Wang *et al.*, *RSC Adv.*, 2019, **9**, 37195–37200.

The authors regret that the contact email address for the corresponding authors was incorrect in the original article. The correct email addresses are as shown here.

The Royal Society of Chemistry apologises for these errors and any consequent inconvenience to authors and readers.

## Supplementary Material

